# Structured therapeutic exercise program effect on PROM and muscle mass in dogs with osteoarthritis: a prospective clinical study

**DOI:** 10.3389/fvets.2026.1836746

**Published:** 2026-07-06

**Authors:** Magdaléna Török, Mária Dvoranová, Tomáš Lipták, Aladár Mad'ari, Mária Kuricová

**Affiliations:** University of Veterinary Medicine and Pharmacy in Košice, Košice, Slovakia

**Keywords:** degenerative joint disease, dog, goniometry, limb circumference, physiotherapy

## Abstract

**Background:**

Degenerative joint disease, namely osteoarthritis, represents a major cause of chronic pain and functional limitation in dogs. Although rehabilitation is widely incorporated into clinical management, objective data quantifying joint mobility and muscular adaptations following structured exercise protocols remain limited.

**Objective:**

To evaluate changes in passive range of joint motion (PROM) and limb circumference following a five-week therapeutic exercise program in dogs diagnosed with degenerative joint disease.

**Methods:**

Twenty-one client-owned dogs underwent a standardized rehabilitation protocol consisting of three sessions per week. Goniometric measurements of appendicular joints and limb circumference assessments were performed before and after intervention. Deviations from published reference midpoints were calculated, and improvement in joint motion was defined as reduction in deviation regardless of direction of absolute change. Morphometric parameters were also recorded. Joints were analyzed individually to describe distribution of changes across the cohort.

**Results:**

Across most joints, extension movements were associated with a higher proportion of PROM increases, whereas flexion showed a more mixed pattern with frequent decreases or unchanged values. The most pronounced response was observed in the hip joint. Pelvic limb musculature showed greater circumference than thoracic limb musculature, with the largest mean increase in circumference recorded in the thigh region. Body weight and trunk circumferences remained largely stable during the intervention period. Considerable inter-individual variability in response to therapy was observed.

**Conclusion:**

A structured exercise-based rehabilitation program was associated with measurable changes in goniometry and muscle circumference in dogs with secondary degenerative joint disease. These findings support the integration of targeted therapeutic exercise into multimodal conservative management strategies for orthopedic patients.

## Introduction

Osteoarthritis (OA) is one of the most common chronic diseases in dogs and represents a significant cause of pain and lameness. If left untreated, it often leads to progressive deterioration of mobility and quality of life. Although OA cannot be cured, long-term disease management can be successful when combining pharmacological pain control, weight management, nutritional support, and rehabilitation (1–3).

Osteoarthritis is a degenerative disorder affecting the entire joint, including cartilage, subchondral bone, synovium, and the joint capsule. Pathological processes involve degradation of the cartilage extracellular matrix, synovitis, and osteophyte formation. Inflammation of the synovial membrane is considered a significant contributor to pain development and progression of degeneration. Reduced activity associated with OA results in decreased joint flexibility, limited range of motion, and muscle atrophy, which may contribute to periarticular changes and further functional decline (1, 4, 5). Regular, controlled physical activity supports muscle tone and joint function, whereas excessive loading may exacerbate clinical signs (6). Goniometry with measurement of passive range of motion (PROM) serves not only as a therapeutic technique but also as an objective assessment tool, with goniometric measurement allowing quantification of PROM in degrees (7). Limb circumference measurement may provide an additional indicator of functional limb use, although inter-observer variability has been reported (8). Despite the widespread use of rehabilitation protocols in clinical practice, objective data describing changes in PROM following structured therapeutic programs remain limited. Systematic evaluation of PROM changes may contribute to a better understanding of the effectiveness of rehabilitation interventions in dogs with secondary osteoarthritis. The objective of this prospective clinical study was to assess changes in passive joint range of motion and muscular circumference following a standardized therapeutic exercise protocol in dogs with secondary osteoarthritis.

## Materials and methods

Dogs diagnosed with osteoarthritis based on clinical and radiographic findings were prospectively included in the study. Dogs presented with different degrees of osteoarthritis severity. The therapeutic exercise protocol used in this study represents a standard therapeutic program routinely offered to canine patients diagnosed with osteoarthritis in our clinical practice. No experimental modifications were introduced for research purposes. Dogs were enrolled consecutively as part of routine clinical care. Only patients that completed the full 5-week rehabilitation program (three times a week) and had complete pre- and post-intervention measurements were analyzed. All owners provided written informed consent for the use of anonymized clinical data for research purposes. All procedures performed in this study were part of routine clinical management and standard rehabilitation care for dogs diagnosed with osteoarthritis. No experimental interventions, invasive procedures beyond standard care, or deviations from accepted clinical practice were introduced for research purposes. According to institutional and national regulations, formal ethical committee approval was therefore not required for this observational clinical study.

At the initial visit, each dog underwent a standardized clinical orthopedic assessment. Dogs were included in the study based on predefined inclusion and exclusion criteria. All included dogs underwent bilateral evaluation of all relevant appendicular joints, regardless of the clinically more affected side. Dogs were eligible for inclusion if they met the following criteria: confirmed diagnosis of OA affecting at least one appendicular joint, based on clinical and imaging findings, presence of chronic orthopedic lameness attributable to OA, age ≥ 2 years, owner consent for participation in the study. Dogs were excluded if they presented with acute orthopedic trauma unrelated to OA, neurological disorders affecting gait or limb use, systemic diseases influencing mobility (e.g. neoplasia, severe cardiovascular disease), previous surgical intervention of the evaluated joints within 2 years.

Lameness severity was subjectively evaluated during walking and trotting examination by an experienced clinician. Pain associated with osteoarthritis was assessed based on clinical orthopedic examination, including pain response during joint palpation and manipulation, joint stiffness, reduced range of motion, and functional mobility impairment. Because the study was designed as a prospective observational clinical study reflecting routine clinical practice, no formal pain scoring system was used as a primary study outcome measure. However, clinical assessment of osteoarthritis-associated pain and mobility impairment routinely incorporated subjective orthopedic examination together with owner-reported functional evaluation, including LOAD (Liverpool Osteoarthritis in Dogs) assessment when available as part of routine clinical management (9).

Some dogs were already receiving conservative osteoarthritis treatment prescribed prior to enrolment, including non-steroidal anti-inflammatory drugs (NSAIDs) or joint-supportive therapy. Analgesic treatment prior to inclusion was therefore not standardized, and no additional analgesic protocol was introduced specifically for study purposes before baseline assessment. During the rehabilitation period, dogs continued to receive routine clinical management as determined by the attending clinician. No dog developed clinically significant worsening requiring discontinuation of the rehabilitation program during the study period.

### Goniometric measurement and limb circumference

At the beginning, each dog underwent standardized clinical assessment including goniometric measurement of joint range of motion (PROM) and limb circumference evaluation. PROM was measured using a standard universal metal goniometer with the dog positioned in lateral recumbency. All appendicular joints were assessed sequentially in each lateral recumbency on one side and then the contralateral side. Each joint was measured three consecutive times by an experienced clinician and the mean value of the three measurements was recorded.

Limb circumference was measured in a standing position using a flexible measuring tape. Measurements were obtained at standardized anatomical locations defined as the mid-portion of each limb segment: mid-humerus (between the tuber scapulae and olecranon), mid-antebrachium (between the olecranon and carpus), mid-femur (between the greater trochanter and patella), and mid-tibia (between the stifle joint and calcaneus). These landmarks were selected based on palpable bony reference points to ensure repeatability of measurements. The measuring tape was maintained in a horizontal position around the limb. Each measurement was performed three times, and the mean value was recorded.

### Body weight and body circumference

Body weight was recorded using a calibrated veterinary scale. Thoracic circumference was measured using a flexible measuring tape at the level immediately caudal to the scapulae. Abdominal circumference was measured at the level of the umbilicus. The measuring tape was maintained in a horizontal position around the body. Each measurement was performed three times, and the mean value was recorded.

### Therapeutic exercise protocol

The therapeutic exercise program was standardized and structured into three sequential phases: warm-up with active stretching, structured therapeutic exercise, and cool-down with passive stretching.

The rehabilitation protocol used in this study was developed based on commonly recommended physiotherapeutic principles for canine osteoarthritis described in previous veterinary rehabilitation studies and textbooks (1, 10–12). The selected exercises were aimed at improving joint mobility, muscle mass maintenance, proprioception, postural control, and functional limb use while minimizing excessive joint loading. The protocol incorporated active mobilization, balance training, cavaletti exercises, and controlled strengthening exercises, which have previously been described as beneficial components of multimodal rehabilitation programs in dogs with osteoarthritis and orthopedic disease. In addition, the protocol reflects the routine rehabilitation approach commonly used in our clinical practice for dogs with osteoarthritis undergoing conservative management.

Each session lasted approximately 60–90 min and was performed three times per week. The rehabilitation program was individualized; however, exercise progression followed a predefined level-based protocol. Advancement to a higher difficulty level was allowed only when the dog demonstrated correct execution of the current level without signs of fatigue, pain, or compensatory movement patterns. Exercise selection and progression were supervised and adjusted by an experienced rehabilitation clinician according to patient tolerance, mobility, pain response, and ability to correctly perform the exercises.

#### Warm-up and active stretching

The warm-up phase was standardized in terms of duration and sequence, while individual exercise selection was kept consistent across patients. The total warm-up duration ranged from 10 to 15 min and consisted of locomotor activation, active stretching, and controlled mobility exercises.

Locomotor activation included cavaletti walking, slalom walking, and figure-of-eight exercises performed for 5–8 min.

Active spinal mobilization was performed in a standardized sequence and included cervical, thoracic, lumbar, and lateral flexion movements (5 directions, 3 repetitions each). Active limb stretching followed, with 2–3 s holds performed three times per limb.

A sit-to-stand exercise was performed five times to activate hindlimb musculature and core stabilization. The warm-up concluded with controlled directional changes (three turns per side) and 1 min of slalom walking.

#### Structured exercise

The structured exercise phase included box exercises, balance training, cavaletti rails, dome exercises, and cone-based coordination training. All modalities were performed according to a three-level progression system with predefined duration and repetition schemes.

#### Box exercises

Three postural positions were used (forelimbs elevated, quadrupedal stance, hindlimbs elevated). Exercise duration progressed from 30 s to 1.5 min per repetition (3 sets × 3 repetitions), with 1-min rest intervals. Progression included introduction of active stretching (level 2) and unstable surfaces (balance disc, 60 cm diameter) at level 3.

#### Balance exercises

Balance exercises were designed to improve neuromuscular control and core stabilization. Training began with static forelimb placement on a balance disc, followed by progression to dynamic pivoting movements and dual-disc stance exercises.

Exercise intensity was structured in a three-level progression system. At the initial level, exercises were performed for 2 min. At intermediate and advanced levels, duration was progressively increased up to 8–10 min per session depending on patient tolerance.

Progression between levels was based on successful completion of the previous level without signs of fatigue, loss of balance, or compensatory movement patterns.

#### Cavaletti training

Cavaletti exercises were used to improve proprioception, joint range of motion, and limb coordination. Pole height and arrangement defined difficulty:

Level 1: poles on ground (3 × 1 min)

Level 2: poles at carpal height (3 × 3 min)

Level 3: elevated and diagonal placement (3 × 5 min)

#### Dome training

Dome-based training targeted proprioception and limb awareness. Progression included single-limb placement and four-limb stance with or without active limb lifting. Duration ranged from 30 s to 5 min.

#### Cone exercises

Slalom walking and figure-of-eight movements were performed for coordination and limb muscle activation (5 repetitions per variation).

#### Cool-down and passive stretching

The cool-down phase aimed to gradually decrease cardiovascular activity, facilitate metabolic waste removal, and promote psychological and muscle relaxation. Dogs were leash-walked at decreasing speed until reaching a slow walking pace. Passive stretching was then performed along the anatomical axis of each limb, including controlled flexion and extension of all major joints.

### Statistical analysis

Statistical analysis was performed using descriptive statistics. PROM changes were calculated as post-treatment minus pre-treatment values. Deviation from reference PROM midpoint was calculated as the absolute difference between measured value and reference value. Reference PROM midpoints were derived from published normal ranges according to Millis and Levine (12). Improvement was defined as a reduction in deviation. Joints were analyzed individually to describe the distribution of changes across the cohort. Because multiple joints originated from the same individuals, the observations could not be considered fully statistically independent. Therefore, inferential statistical comparison at the joint level was not performed in order to avoid pseudoreplication and overinterpretation of the data. The study was designed primarily as a descriptive observational study focused on rehabilitation-associated trends in goniometric and morphometric measurements across evaluated joints and limb regions. Data are presented as mean ± standard deviation (SD) and median values where appropriate. For patient-level morphometric variables, descriptive 95% confidence intervals (CI) were calculated for mean pre–post changes.

## Results

The study cohort consisted of 21 dogs diagnosed with osteoarthritis. The population included 12 males (57.1%) and 9 females (42.9%). Ten dogs (47.6%) were neutered. The mean age was 7.25 ± 2.38 years (range 2.5–11), with a median age of 7.0 years (IQR 6.0–9.0). The distribution of body size categories was as follows: 66.7% large dogs (*n* = 14), 14.3% medium-sized dogs (*n* = 3), and 19.0% small dogs (*n* = 4). The study population consisted of dogs of various breeds, with the most represented being German Shepherd (*n* = 4), Labrador Retriever (*n* = 3), Central Asian Shepherd (*n* = 3), and Standard Poodle (*n* = 3). Additional breeds included mixed-breed dogs (*n* = 3), Dachshund (*n* = 1), Bohemian wire-haired Pointing griffon (*n* = 1), and Alpine Dachsbracke (*n* = 1).

Dogs were not formally stratified according to breed, body weight category, affected joint, or severity of osteoarthritic lesions due to the relatively small sample size and heterogeneity of the clinical population. The study was designed as a descriptive observational study reflecting a real-world rehabilitation population, and therefore dogs with different signalment characteristics and different affected joints were included and analyzed descriptively.

All enrolled dogs were diagnosed with secondary osteoarthritis resulting from joint incongruity or trauma. The hip was the most frequently affected joint (42.9%, *n* = 9), followed by the elbow (28.6%, *n* = 6), the stifle (19.0%, *n* = 4), the digital joints (4.8%, *n* = 1), and the tarsus (4.8%, *n* = 1). Each joint type was evaluated bilaterally, resulting in 42 evaluated joints per movement (21 dogs). The pre- and posttreatment PROM changes are shown in Table 1.

**Table 1:** Changes in PROM (mean ± SD) relative to reference midpoints (*n* = 42 joints per movement) and percentage of joints that improved toward the reference value vs. those that worsened or unchanged, i.e., whose values deviated further from the reference or did not change.

Joint type	Movement	PROM change (°)	Deviation pre (°)	Deviation post (°)	Improved (%)	Worsened (%)	Unchanged (%)
Shoulder	Flex	−1.68 ± 10.95	10.00	7.00	50.0	9.5	40.5
Shoulder	Ext	7.50 ± 18.36	19.00	13.00	38.1	28.6	33.3
Elbow	Flex	−2.84 ± 13.47	15.00	10.00	50.0	19.0	31.0
Elbow	Ext	1.00 ± 7.45	10.00	9.50	47.6	19.0	33.3
Carpus	Flex	−3.65 ± 10.14	7.50	7.50	28.6	19.0	52.4
Carpus	Ext	7.03 ± 13.58	15.00	5.00	59.5	19.0	21.4
Hip	Flex	2.83 ± 11.77	33.50	30.50	28.6	28.6	42.9
Hip	Ext	7.69 ± 8.78	19.50	12.50	64.3	14.3	21.4
Stifle	Flex	−2.19 ± 7.79	10.00	11.00	40.5	23.8	35.7
Stifle	Ext	2.97 ± 8.58	10.00	6.00	33.3	26.2	40.5
Tarsus	Flex	−4.11 ± 11.81	15.00	17.50	35.7	38.1	26.2
Tarsus	Ext	1.94 ± 13.63	10.00	10.00	54.8	19.0	26.2

Median difference from reference value (deviation) before (pre) and after (post) completion of the structured exercise program.

PROM measurements were compared with published reference (12). Improvement was defined as a reduction in deviation from the reference PROM range, regardless of whether the change was positive or negative in absolute value. Standard deviations were relatively high across several movements, reflecting substantial inter-individual variability within the cohort. A positive change (increase in degrees) indicated an increase in joint mobility, whereas a negative change reflected a reduction in joint motion. Joints demonstrating no measurable change in PROM were interpreted as unchanged. The most pronounced response to rehabilitation was observed in hip joint extension, where improvement was recorded in 64.3% of evaluated joints. A higher proportion of improvement was observed in extension movements compared to flexion across most joint types. Flexion changes were generally smaller and demonstrated greater variability across joint types, with higher standard deviations and less consistent improvement patterns.

Body weight showed a slight overall decrease following the rehabilitation program (mean change −0.36 ± 1.31 kg) (Table 2). Thoracic circumference remained relatively stable, while waist circumference demonstrated a small overall reduction. Individual responses varied, with some dogs showing reductions in body weight and waist circumference, whereas others maintained or slightly increased these values. No clinically relevant changes in body weight or trunk circumferences were observed over the 5-week intervention period.

**Table 2:** Changes in body weight and morphometric measurements (*n* = 21) before and after treatment.

Parameter	Pre mean ±SD	Post mean ±SD	Mean change ±SD	95% CI of mean change
Weight (kg)	34.75 ± 18.76	34.39 ± 18.00	−0.36 ± 1.31	−0.96 to 0.24
Thoracic circumference (cm)	76.87 ± 16.83	76.74 ± 16.11	−0.13 ± 1.78	−0.94 to 0.68
Waist circumference (cm)	61.35 ± 14.58	61.08 ± 13.79	−0.27 ± 2.75	−1.52 to 0.98

Muscle mass changes were evaluated based on limb circumference measurements (Table 3). An increase in circumference was considered indicative of improved muscle mass, whereas a decrease was interpreted as muscle loss. Overall, positive changes in muscle mass were more frequent in pelvic limb musculature compared to thoracic limb musculature. The greatest proportion of positive changes was observed in thigh and crus musculature. Quantitatively, the largest mean increase was recorded in the thigh, followed by the crus and shoulder. Variability was highest in thigh measurements, reflecting heterogeneous individual responses.

**Table 3:** Changes in limb circumference measurements after treatment.

Muscle group	Mean change ±SD (cm)	Increased *n* (%)	No change *n* (%)	Decreased *n* (%)
Shoulder	+0.64 ± 1.29	17 (40.5%)	21 (50.0%)	4 (9.5%)
Antebrachium	+0.13 ± 0.87	13 (31.0%)	23 (54.8%)	6 (14.3%)
Thigh	+0.90 ± 1.94	22 (52.4%)	13 (31.0%)	7 (16.7%)
Crus	+0.71 ± 1.30	22 (52.4%)	14 (33.3%)	6 (14.3%)

## Discussion

Rehabilitation is a relatively new sub-specialty in veterinary medicine. The first reports date back to the 1980s, and awareness of its benefits continues to expand (13). In recent years, an increasing number of studies have focused on physiotherapy and functional outcome assessment in canine osteoarthritis (OA), highlighting the growing importance of rehabilitation in clinical practice (11, 14–17). In the present study, we evaluated a therapeutic exercise-based rehabilitation program in 21 dogs diagnosed with osteoarthritis and its effects on PROM.

Bockstahler et al. (18) compared kinematic parameters in dogs with hip joint OA. Their study included 10 dogs with a mean age of 6.95 years. Using motion capture analysis, they calculated maximal hip flexion and extension and found that OA patients exhibited reduced maximal hip extension. When stepping over low obstacles, dogs significantly increased maximal stifle flexion as well as maximal tarsal flexion during motion, so the maximal hip extension was not comparable to maximal PROM. Hip OA was shown to induce complex gait adaptations involving multiple joints beyond the affected hip joint itself. Low obstacle walking was also included in our rehabilitation protocol.

In the present study, hip extension demonstrated the most pronounced response to rehabilitation, with improvement observed in 64.3% of evaluated joints. In contrast, hip flexion improved in 28.6% of joints and showed greater variability. These findings suggest that therapeutic exercise may have a stronger impact on restoring extension capacity of the hip joint, which is commonly reduced in dogs with osteoarthritis. These results are in line with more recent studies investigating UWTM-related changes in joint mobility, which report that structured physiotherapy can lead to measurable improvements in joint range of motion in dogs with OA (17).

Similarly, Dini et al. (19) investigated the effects of physiotherapy in dogs with pelvic limb OA. Two groups of six dogs were followed. The first group received active exercise, passive stretching, magnetotherapy, and electrostimulation, whereas the second group underwent active exercise, underwater treadmill therapy, and thermotherapy. Both groups demonstrated increased PROM in the hip and stifle joints, significant increases in pelvic limb muscle mass, and reduced pain levels.

Cuervo et al. (20) compared the effect of platelet-rich plasma injection with a group that underwent physiotherapy following injection. The rehabilitation program aimed to increase joint PROM, pelvic limb muscle mass, and physical endurance. Exercises were performed once daily and consisted of warm-up, sit-to-stand exercises, limb lifting with subsequent walking, treadmill walking, and cool-down. Improvement was reported in both groups; however, the rehabilitation group did not show functional deterioration during the 6-month follow-up period, whereas the non-rehabilitation group demonstrated deterioration after 3 months.

Our study focused specifically on therapeutic exercise and demonstrated its effects on PROM, with improvement observed in 38.9% of evaluated flexion movements and 49.6% of extension movements across the cohort. The comparisons between the assessed PROM values and reference data from Millis and Levine (12) are based on non-breed-specific normative values, which should be considered when interpreting the results, as breed-related anatomical differences may influence joint range of motion.

Dycus et al. (10) emphasized that active and passive range of motion exercises combined with stretching are essential for minimizing joint pain and improving mobility. The impact of OA on joint motion is joint-specific: dysplastic hips tend to lose extension but not flexion, dysplastic elbows primarily lose flexion, and osteoarthritic stifles lose extension. Our findings are consistent with these observations, as the majority of patients showed improvement across hip, stifle, and elbow joints. These results support the importance of therapeutic exercises, massage, and both passive and active stretching as integral components of multimodal management in osteoarthritic patients with adequately controlled pain. More recent literature further supports the role of structured physiotherapy and multimodal rehabilitation approaches in improving functional outcomes in dogs with OA (11, 21).

Rehabilitation should be considered as part of a broader multimodal management strategy for canine osteoarthritis. In addition to therapeutic exercise, clinical management commonly includes body weight optimization, controlled activity modification, pharmacological analgesia, nutritional support, and environmental adaptations aimed at improving patient comfort and mobility. Non-steroidal anti-inflammatory drugs (NSAIDs) remain among the most commonly used pharmacological options for long-term pain management in dogs with OA, although adjunctive therapies such as gabapentin, amantadine, or joint-supportive nutraceuticals may also be considered in selected patients.

Additional rehabilitation modalities, including hydrotherapy, underwater treadmill therapy, massage, thermotherapy, laser therapy, and neuromuscular stimulation, may further contribute to improved mobility and quality of life in osteoarthritic patients. Environmental modifications, such as non-slip flooring, controlled exercise routines, and reduction of excessive joint loading, may also play an important role in long-term management. Because osteoarthritis represents a progressive chronic disease, successful treatment generally requires an individualized multimodal approach combining pain control with maintenance of functional mobility and muscle condition (2, 11, 15, 22, 23).

Although the exercises applied are not technically demanding, correct methodological execution in accordance with anatomical and physiological principles is crucial. Obesity in dogs is considered a significant risk factor for the development and progression of osteoarthritis (24). Impellizeri et al. (25) investigated the relationship between weight loss and lameness reduction. Dogs were weighed, assigned a body condition score (BCS), and evaluated for pelvic limb lameness and hip function. Lameness severity was assessed using both a numerical rating scale and a visual analog scale. Dogs were fed a calorie-restricted diet providing 60% of the maintenance energy requirement. Assessments were repeated midway and at the end of the 6-month weight-loss period. Dogs lost between 11% and 18% of their initial body weight, and body weight, BCS, and pelvic limb lameness severity significantly decreased by the end of the study.

Marshall et al. (26) similarly demonstrated that body weight reduction results in decreased clinical signs of OA, with improvement observed in dogs that reduced body weight by 6.10%−8.85%. They evaluated dogs undergoing both weight reduction and rehabilitation; however, interpretation of results was challenging due to the combined interventions. The authors concluded that simultaneous management of obesity and osteoarthritis effectively reduces disability in dogs with OA (27). Mlacnik et al. (24) also reported that overweight dogs with gait abnormalities due to osteoarthritis showed clinical improvement when weight reduction was combined with physical therapy.

In our study, comparable trends were observed. Overweight patients that achieved weight reduction generally demonstrated favorable clinical trends during rehabilitation. During the 5-week study period, we recorded an average body weight reduction of 2.3%, which may have contributed to the observed functional improvements, although the relatively short intervention period likely limited the magnitude of weight-related effects.

Given the heterogeneity of the study population, including variability in affected joints, the statistical approach was primarily descriptive. Joints were analyzed individually to capture joint-specific responses to rehabilitation. Because multiple joints originated from the same individuals and the sample size was relatively small, inferential statistical analysis at the joint level was not considered appropriate. Therefore, the results are presented descriptively to reflect response patterns within this clinically heterogeneous population.

Inter-limb symmetry is commonly used as an indicator of functional improvement in orthopedic patients; however, interpretation of symmetry in joint range of motion should be approached with caution. Breed-related and individual variability in physiological joint mobility, together with the absence of universally applicable reference values, limits the interpretation of inter-limb differences as indicators of functional normalization. In the present study, inter-limb comparisons were therefore included only as descriptive parameters characterizing the distribution of joint mobility within individual animals.

A limitation of the present study is the absence of a control group, which restricts the ability to definitively attribute the observed improvements solely to the rehabilitation intervention. The inclusion of a randomized control group would have strengthened the study design and allowed for more robust comparison of treatment effects. However, withholding rehabilitation from dogs diagnosed with osteoarthritis may raise ethical concerns, particularly given that physiotherapy is increasingly considered an important component of multimodal management aimed at reducing pain and improving function. Therefore, all enrolled patients received active treatment. Despite this limitation, the consistent changes observed in joint range of motion across multiple joints suggest a potential beneficial effect of the therapeutic exercise program. Future studies should aim to include randomized controlled designs to further validate these findings and to better isolate the contribution of rehabilitation interventions.

Another limitation of the present study is the relatively small sample size (*n* = 21), which may reduce the statistical power and limit the robustness of the conclusions. A formal power analysis was not performed prior to the study, and therefore the study may be underpowered to detect smaller effects. In addition, the study population was heterogeneous with respect to affected joints (hip, stifle, elbow), as well as body size and age of the dogs. This variability may limit the generalizability of the findings and complicate the interpretation of joint-specific responses to rehabilitation. On the other hand, such heterogeneity reflects the clinical presentation of canine osteoarthritis in everyday practice, where patients differ considerably in signalment and disease manifestation. The present results should therefore be interpreted as representative of a real-world clinical population rather than a strictly controlled experimental cohort.

Due to the pre–post study design without a control group, it is not possible to conclusively attribute the observed changes in PROM and limb circumference solely to the rehabilitation program. Although efforts were made to standardize the measurement procedure, some degree of intra- and inter-observer variability cannot be excluded, but this was not assessed. Therefore, the results should be interpreted with caution, and future studies incorporating controlled and randomized designs are needed to better isolate the specific effects of rehabilitation interventions.

A limitation of the study is the absence of standardized analgesic management and formal pain scoring, which may have contributed to inter-individual variability in functional response to rehabilitation.

An additional limitation is related to the statistical approach used in this study. The analysis was primarily descriptive, which limits the ability to draw formal inferential conclusions regarding treatment effects. Given the heterogeneity of the study population and the fact that multiple joints were assessed within individual dogs, treating joints as independent observations would have introduced pseudoreplication. Due to these methodological constraints and the relatively small sample size, more advanced statistical analyses were not used. Future studies with larger and more homogeneous cohorts should apply statistical models that account for repeated measurements within individuals, such as mixed-effects models, to provide more robust evaluation of treatment effects.

## Conclusion

This study has several limitations, including the absence of a control group, a relatively small sample size, and a short follow-up period. In addition, the use of descriptive statistics without inferential testing limits the ability to assess statistical significance.

Given the lack of a control group, it is not possible to clearly attribute the observed changes solely to the intervention, as they may also reflect natural variability, the effects of ongoing NSAID treatment, changes in daily activity, or measurement-related factors.

Nevertheless, changes in PROM and limb circumference were observed during the rehabilitation period. Extension movements appeared to show a higher proportion of PROM increases, whereas flexion demonstrated a more variable pattern, with both increases and decreases across joints. Due to the observational design of the study and the absence of a control group, a causal relationship between the rehabilitation program and the observed changes cannot be established. PROM and limb circumference were selected as predefined objective outcome measures because the aim of the study was to evaluate changes in joint mobility and muscle condition rather than pain severity. These findings should therefore be interpreted with caution, and further controlled studies are needed to clarify the specific effects of structured therapeutic exercise in dogs with osteoarthritis.

## Data Availability

The original contributions presented in the study are included in the article/supplementary material, further inquiries can be directed to the corresponding author.
